# Appearance Matters: Neural Correlates of Food Choice and Packaging Aesthetics

**DOI:** 10.1371/journal.pone.0041738

**Published:** 2012-07-25

**Authors:** Laura N. Van der Laan, Denise T. D. De Ridder, Max A. Viergever, Paul A. M. Smeets

**Affiliations:** 1 Image Sciences Institute, University Medical Center Utrecht, Utrecht, The Netherlands; 2 Department of Clinical and Health Psychology, Utrecht University, Utrecht, The Netherlands; 3 Division of Human Nutrition, Wageningen University and Research Centre, Wageningen, The Netherlands; University of Bologna, Italy

## Abstract

Neuro-imaging holds great potential for predicting choice behavior from brain responses. In this study we used both traditional mass-univariate and state-of-the-art multivariate pattern analysis to establish which brain regions respond to preferred packages and to what extent neural activation patterns can predict realistic low-involvement consumer choices. More specifically, this was assessed in the context of package-induced binary food choices. Mass-univariate analyses showed that several regions, among which the bilateral striatum, were more strongly activated in response to preferred food packages. Food choices could be predicted with an accuracy of up to 61.2% by activation patterns in brain regions previously found to be involved in healthy food choices (superior frontal gyrus) and visual processing (middle occipital gyrus). In conclusion, this study shows that mass-univariate analysis can detect small package-induced differences in product preference and that MVPA can successfully predict realistic low-involvement consumer choices from functional MRI data.

## Introduction

Despite the public fear of evil marketers tapping into the consumer’s brain to obtain hidden information, the usage of neuroimaging in consumer research is rising [1]. Most commercial and scientific studies on consumer behavior still employ self-report measures, such as questionnaires, to evaluate products and packages [2], [3]. However, the potential of neuroimaging techniques, especially fMRI, to gain more insight into consumer decision-making processes appears to be high. The pioneering study of Knutson et al. [4] showed that a logistic regression model with neural activation of the insula and the nucleus accumbens could predict the decision to buy a wide range of consumer products with 61% accuracy. Recent fMRI studies employing the general linear model, i.e., traditional mass-univariate analysis, have shown comparable accuracies (e.g., an average of 56% accuracy, [5]) or yielded important insights in the neural underpinnings of consumer choices [6], [7]. For instance, Chib et al [6] showed that there is a common representation of the value of different consumer goods in the brain and Kang et al [7] showed that the computation of both hypothetical and real decisions regarding consumer products involves the same brain areas. In addition, fMRI studies using mass-univariate analyses have focused on specific product characteristics, such as perceived healthiness [8], organically grown logo’s [9] and packaging aesthetics [10].

A promising development in the field of consumer neuroscience is the recent application of multivariate pattern analysis (MVPA) to fMRI data [11]. The advantage of MVPA over the traditional mass-univariate analysis is that they employ associations between voxels (activation patterns) and that they allow for differential responses across individual voxels [12]. It is well-acknowledged that this makes MVPA more sensitive than traditional mass-univariate fMRI analyses [12]. Tusche et al [11] were the first that applied MVPA to fMRI to predict consumer choice. They showed that the hypothetical decision to buy a car could be predicted with 70–82% accuracy by activation patterns in the insular and medial prefrontal cortices.

It is impressive that the choice for a high involvement consumer product, like a car, can be predicted with such high accuracy. However, it is yet unknown how accurately lower involvement every-day decisions, such as those made during grocery shopping can be predicted by MVPA. Therefore, we here assess the accuracy of such techniques for predicting low-involvement consumer decisions. To our knowledge, we are the first to use multivariate pattern analysis (MVPA) to investigate realistic low-involvement package-based consumer choices. A good test-case for this category of decisions is food choice. A first important characteristic of food choice (and low-involvement consumer decisions in general) is that they typically involve choices between relatively homogeneous sets of alternatives with much smaller variations in value. For instance, when someone stands in front of the cookie shelf, the decision to buy cookies, and not another type of snacks, has already been made. The next decision is which kind of cookies to choose from the relatively homogeneous set of alternatives.

A second important characteristic of food choices is that foods are usually packaged. Thus, product characteristics have to be inferred from the package. The impression that a package is intended to create in the mind of the consumer is affected by package characteristics like size, shape, color, images and text. Several studies with unpackaged foods have shown differential neural responses to high and low hedonic foods [13]. However, it is unknown whether this also holds for package-induced differences in preference. One of the current trends in food packaging design is to put emphasis on the healthiness of foods by highlighting nutritional information or health logos. This is believed to be an effective strategy to promote buying, because consumers themselves state that the healthiness of a food is an important motivation for their food choices [14]. However, studies on the association between perceived healthiness and preference have yielded ambiguous results. Some studies suggest that labeling a food as ‘healthy’ decreases behavioral preference for the food [15], while others show no [16], [3] or a positive effect [17], [18]. Therefore, it is not clear whether emphasizing healthiness is helpful in promoting healthy food choices. Another packaging feature which has been shown to affect consumer choices is its aesthetic value [19], [20]. However, the effects of aesthetic value have not been studied in the context of healthy food choice.

In the present study, our first aim was to replicate the brain regions that respond to preferred food packages by using traditional mass-univariate analysis. Our second aim was to investigate to what extent brain activation can predict everyday food choices, with the use of MVPA. We employed a realistic food choice paradigm in which subjects had to choose between two alternatives of the same snack food with different packaging designs. More specifically, the choice was between two alternatives in which the packaging either emphasized the healthiness of the food or not. To gain more insight in the underlying factors of choice and the underlying neural processes, a more exploratory third aim was to assess the predictive value of perceived healthiness as well as other self-report measures involved in food choice (e.g., attractiveness, purchase intention), and to what extent the strongest self-reported predictors of choice correlate with neural activation.

To localize brain regions that respond to preferred food packages and that correlate with the main self-reported predictors of food choice, we used both mass-univariate and state-of-the-art MVPA. Traditional analyses were employed to replicate previous findings. The major contribution of the present study is to apply multivariate pattern analysis to predict choices for food items that vary in their packaging.

## Materials and Methods

### Ethics Statement

The study was approved by the Medical Ethical Committee of the University Medical Center Utrecht and subjects provided written informed consent.

### Subjects

The study comprised twenty women as subjects (age range 19–29; mean age 22.4 years; BMI range 19.2–24.7; mean BMI 21.7 kg/m^2^). Inclusion criteria were being female, having an age between 18 and 30 years, being right-handed and having a healthy weight (BMI between 18.5 and 25 kg/m^2^). Only female subjects were included because research showed that the brains of males and females respond differently to food stimuli [21], [22] and that they differ in their eating behavior [23]. Exclusion criteria were smoking, having a food allergy, having an eating disorder, having a current alcohol consumption of >28 units per week, having a history of medical or surgical events that might significantly affect the study outcome, such as metabolic or endocrine disease, or any gastro-intestinal disorder. We excluded women that followed a diet in the past six months or that had weight fluctuations of more than five kg in the past six months, so as to exclude subjects which might show biases in their food choices for weight management reasons. In addition, women were excluded if they indicated a low (<5 on a nine-point scale) liking for cookies or dairy products in the screening questionnaire. Subjects were recruited with posters at the University Medical Center Utrecht and the adjacent university campus. At the time of recruitment, the aim of the study was not disclosed to the subjects because this could influence their responses. The cover story was that subjects were needed for a study on neural processing of novel foods. They would be required to view and evaluate pictures of novel food products and would receive one of these products as afternoon snack. At the end of the study participants were informed about the actual aim of the study.

### Procedures

The study consisted of two sessions, at least one week apart. During the first session, subjects completed a computer task in which they evaluated the expected tastiness of the stimuli, i.e., the pictures of food packages. During this task, each stimulus was shown for four seconds, after which subjects had to indicate on a nine-point scale how tasty they thought the food product would be. This was done in order to ensure that none of the participants had an aversion towards the stimuli. During the second session, subjects were scanned using functional MRI while performing a food choice task. Subjects were instructed to refrain from eating and drinking (except water) for at least three hours (mean 205±27 min) prior to this session. Before and immediately after scanning, subjects rated hunger, thirst and satiety on a visual analog scale. After scanning, subjects were seated behind a computer to evaluate the stimuli on expected tastiness, perceived healthiness, fat level of the food, attractiveness of the packaging and purchase intention, on a nine-point scale ranging from 1 = very untasty/unhealthy/etc to 9 = very tasty/healthy/etc. Also, subjects indicated the price (€) that they would be willing to pay for the product.

### Stimuli

The visual stimuli consisted of 38 color images of food packages: 19 food products (nine dairy products and ten types of cookies) in two different designs. The packages were designed so that they varied in perceived healthiness. Health is an important self-reported motivation for food choice [14] and health aspects are currently highlighted in advertising and packaging trends. Packaging designs were manipulated by varying the following packaging cues: typography, pictures, textual information and logo’s, resulting in ‘healthy’ and ‘unhealthy’ designs [3], [15]. For healthy packaging alternatives the following packaging cues were used: white, green, blue and low intensity colors, elegant, cursive and slim typography, pictures of ingredients (e.g., grains for cookies), pictures/silhouettes of active persons, textual information (e.g., ‘healthy’) and the Dutch Healthy choice logo. For unhealthy packaging alternatives, the following cues were used: yellow, red, brown and high intensity colors, playful/bold fonts, textual information (e.g., ‘With real butter’). Manipulations were based on research from the Department of Packaging Design and Management of the University Twente in which the association between these packaging cues and perceived (un)healthiness was established in Dutch consumers [24], [25]. An internal report on the association between packaging cues and perceived (un)healthiness is available on request. Stimuli were selected on basis of healthiness ratings in a pretest (n = 15 females who did not participate in the study). The only aspects that were systematically kept identical within a pair of packages were their shape and the photo of the product depicted on the package. Since we were interested in the influence of packaging itself and to avoid effects of familiarity and previous experience with the products, novel packaging designs were used. Table 1 shows that design manipulations were effective in altering the perceived healthiness.

**Table 1:** Mean (SEM) ratings^a^ of the packages designed to look healthy/unhealthy.

	Healthy design	Unhealthy design	P-value for difference
Attractiveness of the package design	5.37 (0.10)	4.74 (0.11)	0.03
Healthiness	5.25 (0.09)	4.09 (0.08)	<0.01
Fat content	5.23 (0.11)	6.14 (0.09)	<0.01
Tastiness (first visit)	6.10 (0.10)	6.03 (0.10)	0.85
Tastiness (second visit, after fMRI scan)	6.26 (0.09)	6.36 (0.09)	0.73
Purchase intention	5.21 (0.10)	4.90 (0.11)	0.19
Price willing to pay (€)	1.31 (0.02)	1.26 (0.03)	0.42

a All measures rated on a 9-point Likert scale ranging from 1 = not at all tasty/healthy/etc to 9 = very tasty/healthy/etc, except for price willing to pay.

### fMRI Task

**Figure 1:**
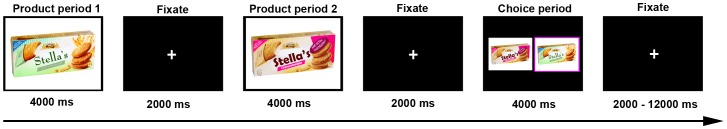
Food choice task trial structure. The first package is the healthy and the second the unhealthy version.

During the functional MRI scan, subjects carried out a food choice task (Figure 1). In this task, subjects made a total of 38 choices between the two package designs, i.e., each of the 19 pairs was presented twice. During each trial the images of the two designs were presented subsequently (product periods, duration 4000 ms each), separated by an inter-stimulus interval of 2000 ms (fixation cross). After that, both alternatives were shown side by side (choice period, duration 4000 ms) and subjects were instructed to indicate with the left or right button of a button box which of the two products they would prefer to eat at that moment. Each trial ended with a fixation cross (random inter-trial interval with duration of 2000–12000 ms). The order of the product presentations and the location of the products during the choice period (left/right) were randomized. In order to make the choices more realistic, subjects were told that one of the trials would be randomly selected and that they would receive the product chosen in that trial as a snack at the end of the study session. In reality, all subjects received the same snack (a commercially available cookie).

### Behavioral Data Analysis

Al self-report ratings (expected tastiness, perceived healthiness, fat level of the food, attractiveness of the packaging, purchase intention and price willing to pay) were normally distributed. Associations between the various self-reported measures were calculated by bivariate correlation analyses performed with SPSS 16.0. Logistic regression analyses were performed to determine associations between the self-reported measures and choice. Since choice pairs (level 1) were nested within participants (level 2) a series of multi-level logistic regression analyses were performed to examine which self-report measures were associated with choosing a package. The dependent variable in these models was the choice for the second stimulus shown (either the package was chosen or not) and the explanatory measures were the difference in ratings between the two packages of the pair (i.e., attractiveness rating of the second image shown (product period 2) minus the attractiveness rating of the first image) for each of the self-reported measures. First, models were constructed with each of the self-report measures as single predictor. After that, models with multiple self-reported measures were constructed. Logistic regression analyses were performed with the statistical software package R (http://www.r-project.org/).

### Image Acquisition and Preprocessing

MRI scanning was performed on a 3 Tesla scanner (Philips Achieva, Philips Healthcare, Best, The Netherlands), equipped with a SENSE head coil. A T1-weighted structural image was acquired at a resolution of 1×1×1 mm (TR = 8.4 ms, total scan duration = 284 s). Functional scans were acquired with a 3D-PRESTO SENSE sequence (TR/TE = 22.5/33 ms, flip angle = 10°, voxel size = 4×4×4 mm, acquisition time of one 3D volume = 607.5 ms) [26]. The total number of volumes acquired differed between subjects because of the random inter-trial interval (range: 1370–1528 volumes). Data were preprocessed and analyzed using the SPM8 software package (Wellcome Department of Imaging Neuroscience, London, United Kingdom, (http://www.fil.ion.ucl.ac.uk/spm/software/spm8/)) run with MATLAB 7.5 (The Mathworks Inc, Natick, MA). Functional images were realigned to the first image of the time series. Functional and structural images were co-registered and normalized (retaining 4x4x4 mm voxels) to MNI space (Montreal Neurological Institute – International Consortium for Brain Mapping) by using linear and nonlinear transformations. Unsmoothed data were used for the multivariate pattern analysis. For the multivariate prediction analysis only data from the first half of the choice trials were used because each choice pair was repeated during the second half of the task. Responses might be biased by post-choice shifts in preferences [27] and thus might not be valid for use in prediction analyses. For the other analyses, all data were used and functional images were smoothed with a Gaussian kernel of 8 mm full width at half maximum.

Data from the first and second product presentation period of the trial were analyzed separately in all analyses because the processes occurring during these periods are not identical. In a sequential binary choice paradigm as used here, the expected value of the first product is evaluated in isolation (the absolute value) whereas the expected value of the second product is evaluated with the first product still in mind (the relative value) [28], [29].

### Traditional Mass-univariate fMRI Data Analyses

#### Subject level analyses

Statistical maps were generated for each subject by fitting a boxcar function to the time series, convolved with the canonical hemodynamic response. Data were high-pass filtered with a cutoff of 128 s. Three conditions were modeled for each trial: the first product presentation period, the second product presentation period and the choice period. For each subject, four separate general linear models were build to perform analyses of the neural activation during the two product presentation periods: (1) To establish the brain regions that respond differently to chosen and not-chosen packages we performed a mean subtraction analysis between chosen and not chosen packages, (2) To identify brain regions of which activation correlates with the self-reported perceived healthiness rating we performed a parametric modulation analysis with perceived healthiness as parametric modulator, (3) To identify brain regions of which activation correlates with the absolute attractiveness we performed a parametric modulation analysis with the self-reported attractiveness as parametric modulator, (4) To identify brain regions in which activation correlates with the relative attractiveness we performed a parametric modulation analysis on the second image period with the relative attractiveness of the second product (i.e., the attractiveness rating of the second package minus that of the first package). In all analyses, the responses during the choice screen (in which the subjects pressed the button for their choice) were modeled but not analyzed. In summary, the subject level analyses yielded seven images for each subject: 1) a contrast image of the chosen versus not chosen packages for the first product presentation period, 2) a contrast image of the chosen versus not chosen packages for the second product presentation period, 3) a contrast image of the parametric modulation of activation by perceived healthiness for the first product presentation period, 4) a contrast image of the parametric modulation of activation by perceived healthiness for the second product presentation period, 5) a contrast image of the parametric modulation of activation by absolute attractiveness for the first product presentation period, 6) a contrast image of the parametric modulation of activation by absolute attractiveness for the second product presentation period, and 7) a contrast image of the parametric modulation of activation by relative attractiveness for the second product presentation period.

### **Group Level Analyses**

To determine which brain regions show differential activation for chosen and not-chosen products, the contrast images in question were entered into a one-sample t-test. To determine the brain regions whose activation is modulated by self-reported healthiness, the contrast images of modulation by healthiness were entered into a one-sample t-test. To determine the brain regions whose activation is modulated by self-reported attractiveness, the contrast images of modulation by absolute and relative attractiveness were entered into a one-sample t-tests. The resulting statistical parametric maps were thresholded at p<0.05 family-wise error corrected for multiple comparisons at the level of a priori regions of interest (i.e., small-volume corrected). Regions of interest were brain areas reported in two studies relevant to food choice: brain regions that respond differentially to highly hedonic versus neutral/bland unpackaged foods [13] and brain regions activated during food choices based on healthiness or tastiness [8]: left inferior frontal gyrus, the bilateral inferior parietal lobule, the bilateral middle temporal gyrus, bilateral superior frontal gyrus, bilateral middle frontal gyrus, right inferior temporal gyrus, bilateral middle occipital gyrus, right culmen and the bilateral putamen, caudate and pallidum. ROI masks were generated using the AAL-atlas [30] as implemented in WFU-pickatlas toolbox [31].

### MVPA

MVPA was used to localize brain regions which contain predictive information. Analyses were performed using the PyMVPA software package [32], in combination with LibSVM’s implementation of the linear support vector machine (http://www.csie.ntu.edu.tw/~cjlin/libsvm/). We used the default configuration, in which the parameter C (trade off parameter between width of the margin and the number of support vectors) is automatically scaled according to the norm of the data for each searchlight.

Trial-wise linearly detrended and z-scored functional scans that were acquired between 3–6 seconds after onset of the product presentation period were averaged to speed up analysis [12]. This timeframe was chosen because the peak of the hemodynamic response is known to occur 4–5 seconds after stimulus onset [33]. This resulted in one average image for the chosen product and one average image for the not-chosen product, for each of the 19 trials.

For both product presentation periods, a whole brain searchlight analysis was performed, which is a method particularly suitable to localize brain regions that contain predictive information [12]. A sphere with a radius of 10 mm was centered at each voxel. With voxel size 4×4×4 mm this results in spheres of 27 voxels, i.e., 27 features. For each sphere, a 19-fold leave-one-out cross-validation was performed with a linear support vector machine to estimate the prediction accuracy of each voxel. Thus, for each sphere the classifier was trained on 18 of the 19 trials. More specifically, a model of the associations between the voxel values and the categories (chosen or not-chosen) in the training trials was constructed. Subsequently, this prediction model was tested on the remaining trial. Accuracy was calculated as the percentage of correctly categorized chosen and not-chosen products in the remaining test trial. For each subject, the searchlight analysis yielded a three-dimensional map of prediction accuracies. Each value of this accuracy map represents the average cross-validated prediction accuracy of the searchlight surrounding that voxel.

To identify brain regions that were predictive of choice across subjects, we performed a t-test as implemented in SPM8 to contrast the accuracy maps of all participants against chance level (50% accuracy) for both analyses. The resulting statistical maps were thresholded at p<0.05 family-wise error corrected for multiple comparisons at the level of ROIs (same as the mass-univariate analysis).

## Results

### Behavioral Results

Table 1 shows that packaging design manipulations were effective in altering perceived healthiness and fat level while keeping expected tastiness constant. The attractiveness of healthy packaging designs was significantly higher. This was expected because visual cues that give a package a healthy appearance are partially overlapping with those that are most preferred. For instance, the colors blue, green and white (bright colors) give a package a healthy appearance, but these are also the colors that are liked most, even across cultures [34], [35], [36], [37].

Attractiveness correlated with several other self-report measures, such as purchase intention (r = 0.65, p<0.001), price willing to pay (r = 0.35, p<0.001), expected tastiness (first session: r = 0.24, p<0.001, second session: r = 0.46, p<0.001) and perceived healthiness (r = 0.15, p<0.001). The results did not differ between the two food categories (cookies and dairy foods); therefore, data from these categories were combined in all subsequent analyses.

Table 2 shows the means of the self-report measures for the chosen and not-chosen packages. Chosen packages were rated as significantly more attractive, tastier and healthier. Purchase intention and the price subjects were willing to pay were also significantly higher for chosen packages. Choices were consistent over repeated presentations: only in 6.8% of the presented choices a different package was chosen the second time. There was no order effect on choice: in 50.7% of the trials the image presented in the second product presentation period was chosen, in 49.3% of the trials the image presented in the first product presentation period.

**Table 2:** Mean (SEM) ratings^a^ for chosen/not-chosen packages.

	Not chosen	Chosen	P-value for difference
Attractiveness	4.17(0.10)	5.81(0.09)	<0.01
Healthiness	4.45(0.09)	4.84(0.09)	<0.01
Fat level	5.75(0.10)	5.63(0.09)	0.43
Tastiness (first visit)	5.97(0.10)	6.14 (0.09)	0.22
Tastiness (second visit, after fMRI scan)	5.93(0.10)	6.64(0.08)	<0.01
Purchase intention	4.45(0.10)	5.57(0.10)	<0.01
Price willing to pay (€)	1.20(0.02)	1.36(0.02)	<0.01

a All measures rated on a 9-point Likert scale ranging from 1 = not at all tasty/healthy/etc to 9 = very tasty/healthy/etc, except for price willing to pay.

The logistic regression analyses with each self-report measure as single predictor showed that the perceived healthiness (parameter estimate ± SEM: 0.17±0.05), attractiveness (0.60±0.07), purchase intention (0.68±0.08), price willing to pay (1.87±0.32) and tastiness post-scan (0.73±0.10) were significantly (positively) associated with food choice (p<0.05). In a model with all self-report measures, only attractiveness, tastiness post-scan and purchase intention remained significant predictors (Table 3), i.e., these measures have an independent component that is associated with choice. This combined model shows that attractiveness has the largest independent component associated with choice. To control for design-category (i.e., healthy or unhealthy design), the analysis was repeated with design category as extra dummy variable. This did not change the results of the logistic regression (Table S1).

**Table 3:** Multi-level logistic regression results: self-report measures associated with food choice.

Model effect	Estimate	Std. Error	Z-value	p	VIF^a^
Fixed effects					
Intercept	0,025	0,131	0,191	0,849	
Attractiveness	0,397	0,078	5,082	<0.001	1.32
Healthiness	0,125	0,103	1,212	0,226	1.93
Fat level	0,026	0,101	0,257	0,797	1.84
Purchase intention	0,230	0,105	2,203	0,028	1.42
Price willing to pay	0,118	0,405	0,293	0,770	1.15
Tastiness session 1	0,132	0,125	1,061	0,289	1.03
Tastiness postscan	0,311	0,125	2,486	0,013	1.33
Random effect(subject)	Variance	SD			
Intercept (level 2)	9,105E-11	9,953E-06			
Log-likelihood model	-179,6				

a VIF = Variance inflation factor is a measure of multicollinearity. A variance inflation factor above 5 indicates high multi-collinearity.

To disambiguate the effects of perceived healthiness and attractiveness on choice we compared the models with attractiveness and perceived healthiness as single predictors with a model with both variables. Whereas the model with both predictors explained significantly more variance than the model with healthiness as single predictor (−2logΛ = 134.5, df = 1, p<0.0001), the combined model did not explain more variance than the model with attractiveness as single predictor (−2logΛ = 0.4 df = 1, p = 0.40). This means that adding healthiness as a variable when attractiveness is already in the model, does not significantly improve the model. Moreover, adding attractiveness to the model decreases the parameter estimate of healthiness from 0.17 to 0.06, while adding healthiness to a model with attractiveness does not affect the parameter estimate of attractiveness. Thus, healthiness does not have an independent component associated with choice while attractiveness does.

Additional likelihood ratio tests were performed to test whether the effects of attractiveness, purchase intention and tastiness varied across participants. This was done by comparing the models with the self-reports treated as fixed effects versus models with random slopes. None of these tests showed a statistically significant improvement of the model. With a likelihood ratio test comparing a model with a fixed intercept to the empty model with a random intercept (subject level 2), we tested whether the intercept was statistically different between subjects. This was also not the case.

### fMRI Results

#### Chosen versus not chosen packages

**Figure 2:**
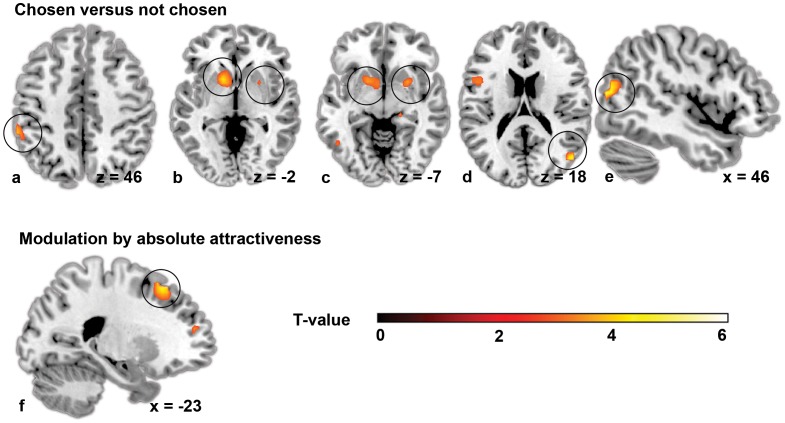
Results from the traditional mass-univariate fMRI analysis. (a–e) Brain regions stronger activated in response to chosen vs. not chosen packages: a) Left inferior parietal gyrus; b/c) left caudate/putamen/pallidum and right putamen; d/e) Border of right middle occipital gyrus and middle temporal gyrus. (f) Brain regions modulated by absolute attractiveness in the first product presentation period: a cluster stretching from the left superior frontal gyrus to the middle frontal gyrus. For visualization purposes, all images are thresholded at T-value >2.86.

The subtraction analysis of chosen vs. not-chosen packages in the second product presentation period showed that activation was stronger for chosen packages in the bilateral striatum (right putamen, left putamen, pallidum and caudate), in the left inferior parietal gyrus, in the middle temporal gyrus and in the right middle occipital gyrus (Table 4, Figure 2). There were no brain regions activated stronger in response to not-chosen packages.

**Table 4:** Peak voxel coordinates^a^ of brain regions stronger activated in response to chosen versus not chosen packages during the second image period in regions of interest.

		MNI-coordinates			Clustersize	
Anatomical label	Side^b^	x	y	Z	(voxels)	Z
Middle temporal gyrus	R	50	−72	18	14	3.77
Putamen	L	−14	12	−2	18	3.60
	L	−22	8	−10		3,17
Caudate	L	−14	16	−2	17	3.22
Pallidum	L	−14	8	−2	5	3.32
Inferior parietal gyrus	L	−58	−40	46	19	3.32
Middle occipital gyrus	R	46	−76	14	4	3.95
Putamen	R	26	8	−10	11	3,30

a Peaks reported are significant at p<0.05 FWE-corrected for the respective ROI.

b L = left hemisphere, R = right hemisphere.

For the first product presentation period, there were no brain regions with a significantly stronger activation in response to chosen packages at P<0.05 FWE-corrected for multiple comparisons at ROI level. Borderline significant clusters were found in the middle frontal gyrus (p = 0.076 FWE-corrected, Z = 3.26, MNI(−26, 20, 58)), the left putamen (p = 0.089 FWE-corrected, Z = 2.64, MNI(−18, 16, 2)) and caudate (p = 0.076 FWE-corrected, Z = 2.73, MNI(−14, 8, 10)).

### Parametric Modulation by Perceived Healthiness & Attractiveness

There were no brain regions in which activation was modulated by perceived healthiness at P<0.05 FWE-corrected for multiple comparisons at ROI level.

We performed a parametric modulation analysis to establish in which brain regions activation is modulated by attractiveness, because the behavioral results showed that attractiveness of the packaging design was strongly associated with choice (see Results section 3.1 Behavioral Results). Brain regions where activation was positively modulated by absolute attractiveness during the first product presentation period were: a cluster in the left superior frontal gyrus (p = 0.002 FWE-corrected, Z = 4.26, MNI(−18, 24, 58) stretching to the left middle frontal gyrus (p = 0.014 FWE-corrected, Z = 4.83, MNI(−22, 24, 54)) (Figure 2). In the second product presentation period, there were no brain regions in which activation was modulated by absolute attractiveness (i.e., the attractiveness rating of the second product). However, there was a borderline significant cluster in the left pallidum (p = 0.092 FWE-corrected, Z = 2.41, MNI(−10, 8, −2)) in which activation was positively modulated by relative attractiveness (i.e., the attractiveness of the second product minus the attractiveness rating of the first product). In neither of the image periods was brain activation modulated negatively by absolute or relative attractiveness.

### Prediction of Food Choice with MVPA

**Figure 3:**
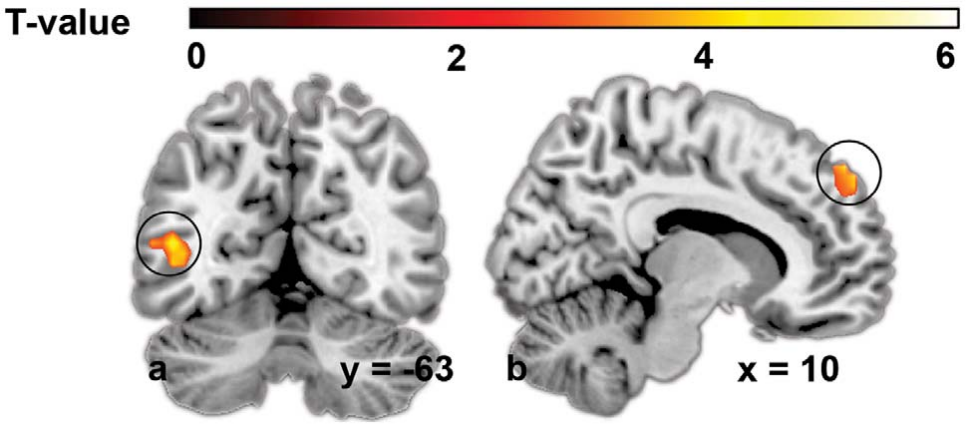
Brain regions predictive of choice. a) left middle occipital gyrus; b) right superior frontal gyrus, medial part. For visualization purposes, images are thresholded at T-value >2.86.

To identify activation patterns that predict choice, MVPA was performed for the first and second product presentation period (Table 5, Figure 3). In the first product presentation period, brain activation patterns in the in the medial part of the right superior frontal gyrus significantly predicted food choice (peak accuracy: 60.0%). For the second product presentation period, activation patterns in the left middle occipital gyrus significantly predicted food choice (peak accuracy 61.2%).

**Table 5:** Brain regions^a^ encoding product choice.

			MNI coordinates			
Brain region	Side^b^	Accuracy^c^ (mean %)	x	y	z	Z-value
First image period:						
Superior frontal gyrus, medial part	R	60,0	10	52	46	3.47
Second image period:						
Middle occipital gyrus	L	61,2	−46	−72	14	4.31

a Peaks reported are significant at p<0.05 FWE-corrected for the respective ROI.

b L = left hemisphere, R = right hemisphere.^ c^ Peak accuracies of clusters are reported.

To ensure the validity of the results, the analyses were repeated with shuffled labels. When samples were randomly provided with a chosen or not chosen label, no statistical significant prediction of food choices could be attained. In addition, in a control region (left fusiform gyrus), which is not usually found in value encoding analyses, we did not find any statistical significant prediction accuracies (maximum accuracy 52.2%). These findings speak against potential methodological concerns such as the over-fitting of noise or insufficient corrections for multiple comparisons.

Table S2 shows results of all analyses for all brain regions (i.e., not only in predefined ROI’s).

## Discussion

This study is the first to investigate both the neural correlates and predictors of choice between two food items that only differ in their packaging.

### Chosen Versus Not Chosen Packages

Our first aim was to replicate brain regions found in previous studies that respond to preferred food packages. The mass-univariate analysis contrasting chosen with not-chosen packages yielded several regions stronger activated in response to chosen packages, among which the bilateral striatum. This finding concurs with our recent meta-analysis [13], which showed that appetizing foods yield a consistently higher activation in the striatum than neutral or bland foods. The studies included in this meta-analysis compared two very different groups of unpackaged foods. Our results show that the same finding holds for package-induced variations in preference within the same product, which presumably are smaller than variations between different products. A recent study of Litt et al. [38] has suggested a role for the striatum in both value and saliency (arousal) computations during food choice. Because we did not include any aversive (negatively valenced but arousing) products we cannot differentiate between these two processes. Thus, the striatal activation in the present study could reflect both value and salience computations.

The finding that clusters in the inferior parietal gyrus and the middle temporal gyrus are more strongly activated during chosen versus not chosen packages, is in line with previous work. Activation of parietal regions during decision-making has been associated with the valuation of different options and a recent meta-analysis has shown that inferior parietal regions were more consistently activated during reward anticipation than during the reward-outcome [39]. Thus, the inferior parietal gyrus activation during the second image period could reflect response selection, since all alternatives are known at that moment. Activation in the middle temporal gyrus during the decision period has previously been found to correlate with stimulus value (but not saliency) [38].

### Prediction of Food Choice with MVPA

#### Brain regions predictive of food choice

Our second aim was to investigate to what extent brain activation can predict food choice. To our knowledge, we are the first to use MVPA to investigate realistic low-involvement consumer choices, such as food choices. Previous studies using traditional analysis techniques investigated the neural correlates of consumer choice (e.g., [8], [4]) and of specific product characteristics (e.g., aesthetics, [10]). However, none of these studies used both traditional analysis and MVPA. It is well-acknowledged that MVPA is more sensitive than traditional mass-univariate fMRI analyses (e.g., [12]). One pioneering study [11] showed the high potential of this novel technique in predicting the hypothetical choices for a high-involvement product (a car). However, until now it was unknown whether this technique could also accurately predict low-involvement consumer choices. Therefore, we assessed how accurately this type of consumer choices, such as those made during grocery shopping can be predicted by MVPA. These are choices in which the differences in preference are assumed to be much smaller than in infrequent high-involvement choices, such as those for a car. By employing a linear support vector machine, we showed that MVPA is also sensitive enough to predict every-day food choices: food choice could be predicted with up to 61.2% accuracy on group level with activation patterns in the right superior frontal gyrus (medial part) and the left middle occipital gyrus. Given that the choice was between two similar foods, which only differed in their packaging design, this can be considered as a high accuracy. Similar accuracies have been found in studies that used traditional mass-univariate analysis methods to predict choice (e.g., [4], [5]). The only other study on consumer choice that utilized MVPA, reported prediction accuracies in the range of 72–80% with patterns in the right middle frontal gyrus, medial frontal gyrus, left orbitofrontal cortex, bilateral dorsal anterior cingulate, bilateral posterior cingulate, and left insula [11]. However, as mentioned above, that study used a very different category of consumer products, namely cars. A decision for a car is probably much more distinct than the low-involvement every-day decision for a snack food: cars are bought less often and less easily than foods. Another difference between the studies is that in the study of Tusche et al [11], all stimuli were familiar. Since we were interested in the influence of the packaging itself and to avoid effects of familiarity and previous experience with the product, we used novel packages as stimuli. Another explanation for the difference in findings is that the question posed in the study of Tusche et al. [11] was to either buy the car or not. Thus, the value of just one car had to be taken into consideration, while in our study subjects had to make a binary choice. That is, preferences for two similar products had to be compared and decisions were thus not based on the absolute value of the products, but on the difference in value between the two options (i.e., the relative value, which will be elaborated on in section 4.2.1).

In the first image period, brain activation patterns in the medial part of the right superior frontal gyrus predicted choice. This is in line with other studies showing that activation in this region correlates with value [38] and willingness to buy [9] during decision-making. Also, interestingly, a cluster at approximately the same coordinates was found to be activated during food choices in which the participants were asked to specifically consider the healthiness of the food [40]. This suggests that this region could be involved in the consideration of product features such as healthiness.

For the contrast between chosen and not-chosen packages as well as for the MVPA, we found involvement of the middle occipital gyrus, although for the prediction analysis the cluster was lateralized to the left and for the mass-univariate analysis to the right. This region is primarily known for its role in visual processing. Differential middle occipital activation for preferred versus not-preferred items has previously been observed in studies with food stimuli, e.g., our meta-analysis showed that across studies, the right middle occipital gyrus is stronger activated in response to highly hedonic versus bland/neutral foods [13]. Although it cannot be ruled out that differential activation in regions involved in color processing such as the middle occipital gyrus and the inferior parietal gyrus, is partly due to color differences between the stimuli [41], [42], an equally likely explanation could be that these findings reflect modulation of visual processing by emotional valence. A recent study showed higher middle occipital activation in response to high- compared to low-energy foods, even though the pictures were matched on visual properties [21]. It has been widely acknowledged that both attention and emotional valence modulate processing of visual stimuli by enhancing neuronal responses at different levels of visual processing, i.e., in early visual processing and in later phases such as recognition [43], [44], [45]. Accordingly, we speculate that the observed activation of visual processing brain areas reflects increased attention to, or emotional valence of preferred packages. Future research should elucidate the exact role of the middle occipital gyrus in emotional valence and value calculation.

### Absolute and Relative Value Calculation

It is important to note that our results show that predictions can be derived from brain activation both during both product presentation periods. However, it should be noted that evaluation processes differ between the two periods: whereas the expected value of the first product is evaluated in isolation (the absolute value), the expected value of the second product is evaluated with the first product still in mind (the relative value). Therefore, the value of the first product serves as a frame of reference against which the second product is weighed. Given our sequential binary choice design one would expect that the neural encoding of chosen and not chosen products is influenced by ordering effects within a trial (i.e., in the first product presentation period the absolute value of the product is computed whereas in the second product presentation period the relative value is computed). There is evidence to suggests that value is more often computed with respect to a reference point, rather than in isolation [29]. This notion is also supported by psychological literature on contrast effects (e.g., [46]), as well as evidence from fMRI and neuronal recording studies which show ordering effects of comparative valuation in brain areas involved in decision-making (e.g., [47], and differential brain regions involved in encoding of absolute and relative value [28]. We therefore argue that evaluation in the second product presentation period is influenced by the preceding stimulus while this is not the case in the first period. Therefore, the brain regions activated during the first image period most likely reflect the absolute value calculation of the product. In contrast, activation during the second image period likely reflects a comparative calculation in which the second product is weighed against the first product, which serves as a reference. Our findings, that the ventral part of the striatum is more strongly activated in response to chosen versus not chosen packages in the second product presentation period, and that activation in the ventral striatum tends to correlate with the relative attractiveness, is in line with findings of De Martino et al. [28]. They also found that ventral striatum activation correlate with the relative value in a buying/selling fMRI paradigm. In addition, although borderline significant, we found that a more dorsal part of the striatum was stronger activated for chosen compared to not chosen packages in the first image period. This is also in line with the finding that a more dorsal part of the striatum correlates with the absolute value [28].

In the fMRI task, subjects indicated their choice when presented with a separate choice screen, shown to them after the first and second product presentation period. This design may complicate the interpretation of the results for the second product presentation period because due to this separate choice screen, we cannot be completely sure whether subjects made their decisions during the second product presentation periods or at the moment they were presented with the choice screen. Nevertheless, we decided to use a separate choice screen in order to avoid biases due to motor responses accompanied with the button press in the evaluation-processes that take place during the product presentation periods. Several studies using a sequential design indicate that the decision process starts during the period when the second stimulus is presented [e.g., [47]]. Thus, although the design of our study does not allow to exactly determine when subjects made their choice (either during the second product period or when presented with the choice screen), we argue that it is likely that decisions were made during the second product period. Even though, evaluation and decision signals were confounded during the second presentation period, we do know that most likely relative value computations are taking place in the second product presentation period. Considering its ecological relevance (e.g., [29]), the differentiation between absolute and relative value computation in the brain is an important topic for future research.

### Difference Mass-univariate Analysis and MVPA

We compared brain activation during chosen and not-chosen products with the use of both traditional mass-univariate analysis and MVPA. The results of the traditional analysis enabled a comparison with results from previous studies. However, this kind of analysis alone, without any validation, is deemed unsuitable to establish a predictive relationship between neural activation and choice [48]. To be able to establish the predictive performance of a technique (either univariate or multivariate), it is required to validate the model, for instance with independent testing and training data sets [12]. Several studies showed that traditional mass-univariate analyses indeed can also yield brain regions predictive of choice, e.g. with the use of cross validation [4] or by using a functional localizer task to identify predictive regions of interest [5]. In our study, we did not find direct overlap between the results of the traditional analysis comparing chosen and not chosen packages and the brain regions predictive of choice in the MVPA. A likely explanation for the lack of overlap between the two techniques is that the underlying calculations are not the same: The main difference between the two methods is that the mass-univariate method tests for differences in level of activation of each voxel separately, while MVPA establishes whether activation patterns (i.e., interactions between multiple voxels) are associated with an outcome measure.

More specifically, differences in brain regions identified by mass-univariate analyses and MVPA likely arise from the fact that (1) different information is taken from the data (i.e., differences in the degree of activation in single voxel vs. interactions between multiple voxels) and (2) differences in the preprocessing and analysis trail associated with (1). The preprocessing and analysis trail of mass-univariate analysis is optimized for detecting spatially extended differences in the degree of activation (i.e., differences in the same direction) while the preprocessing and analysis trail of MVPA is optimized for detection of pattern-based information. For mass-univariate analysis, images are smoothed for improving the signal-to-noise ratio, making the error-distribution more normal and accommodating functional variations between subjects. However, a drawback of smoothing is that it reduces the spatial resolution of the data. In MVPA, on the other hand the focus is on fine-grained activation patterns, therefore data for MVPA are not smoothed. To optimize data for MVPA, z-scoring (setting the mean to zero and standard deviation to 1) is performed to homogenize voxel intensities. Because z-scoring involves scaling all voxel intensities into approximately the same range and removing the mean the difference in activation level between conditions can be diminished. Therefore, a likely explanation of why we find evidence for involvement of the striatum in the mass-univariate analysis but not in the MVPA results is that (although there are differences in average activation for chosen versus not chosen stimuli), the activation patterns in the striatum do not contain (detectable) information that differentiates between conditions. For the regions that we found with MVPA but not with mass-univariate analysis (clusters in middle occipital gyrus, superior frontal gyrus) the opposite holds: although average activation in these regions did not differ for the chosen and not chosen stimuli, activation patterns did contain information that could distinguish between chosen and not chosen stimuli (and could predict this with up to 62,1% accuracy).

The few studies that have employed both methods have also shown different results for mass-univariate analysis and MVPA. For instance, Lee et al [49] found different cortical areas involved in categorical speech processing with mass-univariate analysis and MVPA, and Tusche et al [11] did not find any brain regions significantly associated with consumer choice with a mass-univariate analysis, while the MVPA did differentiate. The fact that mass-univariate and MVPA results do not necessarily concur does not have implications for the interpretation of mass-univariate imaging data to date, i.e., these data remain valid in their own right, but it does highlight that other information can be gained with more sophisticated techniques. It seems likely that for purposes like choice prediction a combination of both mass-univariate analysis and MVPA will become the preferred approach because these two analysis methods complement each other.

### Improving Prediction Accuracy

Prediction accuracy might be further improved by more trials for training and testing. It is hard to determine the optimal number of trials. More training data usually produce a better model and more test samples increase the power of the test for significance of the accuracy [12]. In our analyses, we used cross-validation to maximize the number of data for training. However, performing MVPA with many features (voxels) and relatively few trials entail a risk of over fitting, especially with complex classification models [12]. We avoided this by using a searchlight analysis to reduce the number of features and by employing a simple linear model (linear support vector machine) as classifier. Moreover, the validity of the MVPA results was supported by the finding that the analysis with the shuffled labels did not yield any significant prediction accuracies, and the fact that no significant prediction accuracies were found in a control region (left fusiform gyrus).

### Ventromedial Prefrontal Cortex

The ventromedial prefrontal cortex (vmPFC) has also been implicated in decision-making and activation in this region has been found to correlate with measures of preference (e.g., [6], [4], [38]). In our study, however, there was no significant association between vmPFC activation and the variables of interest, like attractiveness or choice. A possible explanation for this is that ventral prefrontal areas are prone to signal loss due to susceptibility artifacts. Visual inspection of the mean inclusive functional masks showed that there was indeed an (unexpectedly) low signal from ventral prefrontal regions. The specific scanning sequence we used, is known to be very sensitive and has a fast acquisition time [26]. However, the signal in the vmPFC was unexpectedly low. Alternative explanations for not finding vmPFC-involvement could be that we performed no analyses that assessed the trial by trial correlation with values for the food items that would directly replicate the analyses that have identified vmPFC correlations with stimulus or decision value. We did correlate neural activation with self-reported attractiveness. However, this is only one component of stimulus value. A third explanation could be that our design does not allow one to exactly know at what moment subjects decided. As argued previously, it seems most likely that decisions were made during the second product presentation period, although we cannot rule out the alternative explanation that they were made later, i.e., when the ‘decision screen’ was presented. Previous work has shown that vmPFC activation correlates more strongly with stimulus value (WTP) when subjects are engaged in active decision making compared to forced responses [50]. Therefore, a lack of vmPFC correlation with our measures of preference could be due to the unknown timing of the decision.

### Perceived Healthiness and Attractiveness of the Package Design

Our third aim was to investigate how self-reported measures relate to food choice. We found that the attractiveness of the packaging design was the strongest predictor of choice, and that perceived healthiness did not have an independent component associated with choice while attractiveness did. This suggests that, when people choose between two alternatives of a certain type of product with which they are not familiar, the aesthetic value of the package is decisive. That is, an attractive package increases the general preference for the alternative. This could be exploited in promoting healthy eating behavior. Instead of the current strategy of putting emphasis on the healthiness of foods (e.g., by low-fat labels or health logos), it might be more effective to present healthy products in attractive packages. Our study does not allow for determining how attractiveness influences choice, i.e., whether subjects just chose the most attractive package or whether attractiveness influenced the expected value of the food. However, other studies suggest that attractiveness could influence general product preference by triggering positive responses and by increasing the expected quality, luxury and price of products in several consumption domains [51], [52], [53]. As the stimuli we used were unfamiliar to the subjects, it could be that they used attractiveness as a proxy for quality. This would explain why in our study attractiveness is the strongest self-reported predictor of choice. Future research should investigate how attractiveness can influences choice and whether attractive packaging could indeed promote healthy food choices.

We found that brain activation in a region stretching from the left middle frontal gyrus to the superior frontal gyrus was modulated by ratings of packaging attractiveness. This is in line with other studies which found that activation in this region correlates with product preference ratings [54], [4], goal values [55] and willingness to buy [9]. Also, an additional parametric analysis with self-reported purchase intention (Table S3) showed that neural activation in the same region correlated with purchase intention, a measure which correlated to a fair extent (r = 0.65) with attractiveness. From this we speculate that the modulation of activation in the superior frontal gyrus stretching to the middle frontal gyrus reflects a more general preference evaluation which is driven by the attractiveness. This topic deserves more investigation.

The self-reports of healthiness, attractiveness, perceived fat content, purchasing intention and price willing to pay were measured after the choice-task. This has limitations because the act of choosing may induce changes in product preference, such that they better match with their prior decision [27]. However, we nevertheless decided to measure product characteristics after choice, because measuring them before choice focuses attention on them which may also affect their choice. Since we were interested in subject’s spontaneous choice, we chose to avoid ‘priming’ them with the characteristics of interest before their choice. A second reason why these measures were not collected before the choice-task was to limit exposure to the stimuli and avoid a thorough evaluation of the stimuli before the choice because we wanted the evaluation processes to take place during the fMRI task.

### Conclusions

In conclusion, the present study showed that mass-univariate analysis can detect small package induced differenced in preference and that binary food choices could be predicted with an accuracy of up to 61.2% by activation patterns in brain regions previously attributed to healthy food choices (medial superior frontal gyrus) and visual attention processes (middle occipital gyrus). This study confirms the importance of aesthetics in packaging design and suggests that healthy food choices could be promoted by presenting healthy foods in more attractive packages.

## Supporting Information

Table S1 **Multi-level logistic regression results: self-report measures and packaging type associated with food choice.**
(DOC)

Table S2 **Peak voxel coordinates^a^ of brain regions positively modulated by choice,** **attractiveness and brain regions predictive of choice.**
(DOC)

Table S3 **Peak voxel coordinates^a^ of brain regions positively modulated by purchase intention.**
(DOC)
