# Supplementary material for: Appearance Matters: Neural Correlates of Food Choice and Packaging Aesthetics
Source: PLoS One. 2012 Jul 25;7(7):e41738. doi: 10.1371/journal.pone.0041738 (PMC3404976; doi:10.1371/journal.pone.0041738)
Supplement: Table S1 — Multi-level logistic regression results: self-report measures and packaging type associated with food choice. (DOC) [file pone.0041738.s001.doc]

**Table S1 Multi-level logistic regression results: self-report measures and packaging type associated with food choice**

| Model effect | Estimate | Std. Error | Z-value | p | VIF^a^ |
| --- | --- | --- | --- | --- | --- |
| Fixed effects |  |  |  |  |  |
| Intercept | 0,034 | 0,218 | 0,155 | 0,877 |  |
| Packaging type (1=healthy, 0=unhealthy ) | -0,016 | 0,318 | -0,051 | 0,959 | 1.49 |
| Attractiveness | 0,397 | 0,078 | 5,058 | <0.001 | 1.33 |
| Healthiness | 0,126 | 0,107 | 1,178 | 0,239 | 2.09 |
| Fat level | 0,025 | 0,103 | 0,241 | 0,810 | 1.92 |
| Purchase intention | 0,231 | 0,105 | 2,203 | 0,028 | 1.42 |
| Price willing to pay | 0,120 | 0,406 | 0,296 | 0,768 | 1.15 |
| Tastiness session 1 | 0,132 | 0,125 | 1,062 | 0,289 | 1.03 |
| Tastiness postscan | 0,310 | 0,126 | 2,466 | 0,014 | 1.34 |
|  |  |  |  |  |  |
| Random effect (subject) | Variance | SD |  |  |  |
| Intercept (level 2) | 9,305E-11 | 9,763E-06 |  |  |  |
|  |  |  |  |  |  |
| Log-likelihood model | -179,5 |  |  |  |  |

^a^ VIF = Variance inflation factor is a measure of multicollinearity. A variance inflation factor above 5 indicates high multi-collinearity.
