# Supplementary material for: Appearance Matters: Neural Correlates of Food Choice and Packaging Aesthetics
Source: PLoS One. 2012 Jul 25;7(7):e41738. doi: 10.1371/journal.pone.0041738 (PMC3404976; doi:10.1371/journal.pone.0041738)
Supplement: Table S2 — Peak voxel coordinatesa of brain regions positively modulated by choice, attractiveness and brain regions predictive of choice. (DOC) [file pone.0041738.s002.doc]

**Table S2** Peak voxel coordinates^a^ of brain regions positively modulated by choice, attractiveness and brain regions predictive of choice^c^

|  | |  | **MNI-coordinates** | | |  |  |
| --- | --- | --- | --- | --- | --- | --- | --- |
| **Anatomical label** | | **Side^b^** | **x** | **y** | **Z** | **Cluster size (voxels)** | **Z** |
| Parametric modulation by choice – first image period | |  |  |  |  |  |  |
| 1 | Middle frontal gyrus | L | -26 | 20 | 58 | 20 | 3.26 |
| Parametric modulation by choice – second image period | |  |  |  |  |  |  |
| 1 | Middle occipital gyrus | R | 46 | -76 | 14 | 18 | 3.95 |
| 2 | Putamen | L | -14 | 12 | -2 | 62 | 3.60 |
|  | Olfactory | L | -22 | 4 | -14 |  | 3.35 |
|  | Pallidum | L | -6 | 0 | -10 |  | 3.01 |
| 3 | Amygdala/putamen | R | 26 | 4 | -14 | 25 | 3.52 |
| 4 | Inferior parietal gyrus | L | -58 | -40 | 46 | 35 | 3.32 |
|  | Supramarginal gyrus | L | -62 | -40 | 30 |  | 2.84 |
|  | Supramarginal gyrus | L | -62 | -28 | 38 |  | 2.80 |
| 5 | Inferior frontal operculum | L | -50 | 8 | 18 | 12 | 2.95 |
| Parametric modulation by absolute attractiveness – first image period | |  |  |  |  |  |  |
| 1 | Superior frontal gyrus | L | -18 | 24 | 58 | 61 | 4.26 |
| 2 | Posterior cingulum | L | -6 | -48 | 34 | 18 | 2.91 |
| Parametric modulation by absolute attractiveness – second image period | |  |  |  |  |  |  |
| 1 | Supramarginal gyrus | R | 62 | -28 | 38 | 16 | 3.62 |
| 2 | Calcarine gyrus | L | -6 | -92 | 2 | 12 | 3.07 |
| Parametric modulation by relative attractiveness – second image period | |  |  |  |  |  |  |
| 1 | Insula | R | 34 | 20 | -2 | 32 | 3.34 |
| MVPA - second image period | |  |  |  |  |  |  |
| 1 | Middle occipital gyrus | L | -46 | -72 | 14 | 38 | 4.31 |
|  | Middle temporal gyrus | L | -46 | -64 | 10 |  | 4.01 |
|  | Middle temporal gyrus | L | -54 | -60 | 10 |  | 3.39 |
| 2 | Cerebellum | R | 54 | -70 | -34 | 18 | 4.15 |
| 3 | Cerebellum | R | 38 | -48 | -50 | 10 | 3.19 |
| MVPA - first image period | |  |  |  |  |  |  |
| 1 | Superior frontal gyrus, medial part | R | 10 | 52 | 46 | 21 | 3.47 |

^a^ Peaks reported are significant at p<0.005 uncorrected, cluster extent k≥10.

^b^ R = right hemisphere, L = left hemisphere.

^c^ For the modulation analysis with perceived healthiness no significant clusters could be found
