# Supplementary material for: Appearance Matters: Neural Correlates of Food Choice and Packaging Aesthetics
Source: PLoS One. 2012 Jul 25;7(7):e41738. doi: 10.1371/journal.pone.0041738 (PMC3404976; doi:10.1371/journal.pone.0041738)
Supplement: Table S3 — Peak voxel coordinatesa of brain regions positively modulated by purchase intention. (DOC) [file pone.0041738.s003.doc]

**Table S3** Peak voxel coordinates^a^ of brain regions positively modulated by purchase intention

|  | |  | **MNI-coordinates** | | |  |  |
| --- | --- | --- | --- | --- | --- | --- | --- |
| **Anatomical label** | | **Side^b^** | **x** | **y** | **Z** | **Cluster size (voxels)** | **Z** |
| First image period | |  |  |  |  |  |  |
| 1 | Precuneus | L | -2 | -60 | 42 | 99 | 3.57 |
|  | Cuneus | L | -10 | -64 | 30 |  |  |
|  | Middel cingulum | L | -2 | -36 | 38 |  |  |
| 2 | Superior temporal gyrus | R | 42 | -32 | 6 | 11 | 3.36 |
| 3 | Middle frontal gyrus | L | -22 | 28 | 54 | 24 | 3.17 |
| Second image period | |  |  |  |  |  |  |
| 1 | Supramarginal gyrus | L | -62 | -28 | 38 | 13 | 3.26 |
|  | Supramarginal gyrus | L | -62 | -36 | 30 |  | 3.14 |
|  | Inferior parietal gyrus | L | -58 | -28 | 46 |  | 2.70 |
| 2 | Middle occipital gyrus | R | 42 | -72 | 26 | 27 | 3.19 |
| 3 | Middle temporal gyrus | L | -50 | -60 | 6 | 14 | 3.08 |

^a^ Peaks reported are significant at p<0.005, k≥10 uncorrected.

^b^ R = right hemisphere, L = left hemisphere.
